# A Coronavirus Disease 2019 (COVID-19) Patient with Multifocal Pneumonia Treated with Hydroxychloroquine

**DOI:** 10.7759/cureus.7473

**Published:** 2020-03-30

**Authors:** Aveek Mukherjee, Mudassar Ahmad, Douglas Frenia

**Affiliations:** 1 Internal Medicine, Saint Peter's University Hospital/Rutgers Robert Wood Johnson Medical School, New Brunswick, USA; 2 Pulmonary Medicine, Saint Peter's University Hospital/Rutgers University, New Brunswick, USA; 3 Pulmonary Critical Care, Saint Peter’s University Hospital, New Brunswick, USA

**Keywords:** covid-19, 2019-ncov, sars-cov-2 (severe acute respiratory syndrome coronavirus -2), 2019 novel coronavirus, multifocal, pneumonia, hydroxychloroquine, ground-glass opacity, wuhan, cov

## Abstract

After an outbreak in December 2019 in Wuhan, Hubei Province of China, coronavirus disease 2019 (COVID-19) has rapidly become a pandemic. The 2019 novel coronavirus (2019 nCov), now called severe acute respiratory syndrome coronavirus 2 (SARS-CoV-2), causes a wide spectrum of illness and patients with underlying comorbidities have a high mortality. Here we present a 49-year-old male patient with comorbid conditions who presented with fever, cough, myalgia and shortness of breath for five days with likely exposure to a COVID-19 contact. A computed tomography scan of the thorax revealed multifocal bilateral ground-glass lung opacities with areas of subpleural sparing. He tested positive for SARS-CoV-2 by nucleic acid amplification. Hydroxychloroquine therapy was started, and the patient responded favorably with improvement of symptoms. Early diagnosis and self-isolation or quarantine remain key to stemming the tide of the contagion as there is a real risk of the healthcare system being overwhelmed.

## Introduction

Early in December 2019, an outbreak of an acute respiratory disease of unknown etiology was detected in Wuhan, Hubei Province of China [1,2]. A novel beta coronavirus was isolated, initially called the 2019 novel coronavirus (2019-nCov) by the World Health Organization (WHO) [3]. The disease was named as coronavirus disease 2019 (COVID-19) by WHO on 11 February 2020, the same day the Coronavirus Study Group renamed the virus to severe acute respiratory syndrome coronavirus 2 (SARS-CoV-2) [3]. The first case in the United States (US) was diagnosed at an urgent care clinic in Snohomish County, Washington, on 19 January 2020 [4]. The disease has since spread rapidly, prompting the WHO to declare COVID-19 a pandemic on 11 March 2020 [3]. As of 29 March, there are now over 638,000 confirmed cases worldwide in 203 countries or territories, with more than 30,000 deaths and a mortality of 4.7% [3]. SARS-CoV-2 is similar to the severe acute respiratory syndrome coronavirus (SARS-CoV) of 2002 and the middle east respiratory syndrome coronavirus (MERS-CoV) of 2012 which had 10% and 35% mortalities, respectively [1]. Fever, cough, fatigue, sputum expectoration and shortness of breath have been noted to be the most common symptoms, and the disease can present as mild or severe based on the underlying condition of the host [1,2,5]. The diagnosis and management of COVID-19 is still evolving. Here we present a 49-year-old male patient with comorbid conditions and presenting with multifocal COVID-19 pneumonia who we successfully treated with hydroxychloroquine.

## Case presentation

A 49-year-old gentleman with past medical history of hypertension, hyperlipidemia and pre-diabetes presented with fever, cough, myalgia and shortness of breath for five days. The patient is a priest by profession and started having symptoms after Mass five days ago. Initially, the patient noticed generalized tiredness, and then developed a cough, which was initially dry but later became productive of white sputum. The next day, the patient developed a non-remitting fever, as high as 101°F. He visited his primary medical doctor (PMD) the same day and upon evaluation was presumptively diagnosed with influenza and prescribed oseltamivir (Tamiflu) and acetaminophen. Fever as high 102.5°F persisted with associated chills and severe muscle aches. Four days later, the patient experienced shortness of breath during prolonged coughing episodes. He contacted his PMD again and was prescribed doxycycline along with an as-needed albuterol inhaler. However, with continuing high fever, severe weakness and shortness of breath, he presented to the emergency department via emergency medical services ambulance for evaluation.

On examination, the patient was noted to be in moderate distress due to dyspnea. He was febrile to 103°F with tachycardia, tachypnea and elevated blood pressure to 164/96 mmHg. His oxygen saturation was 94% on room air and he exhibited the use of accessory muscles of respiration along with diffuse crackles on lung auscultation. The patient indicated that he had traveled to New York City in the week prior to admission and was probably exposed to a sick contact during his last Mass. He is a lifetime non-smoker with no alcohol abuse and lived with three other priests who were healthy.

**Figure 1:**
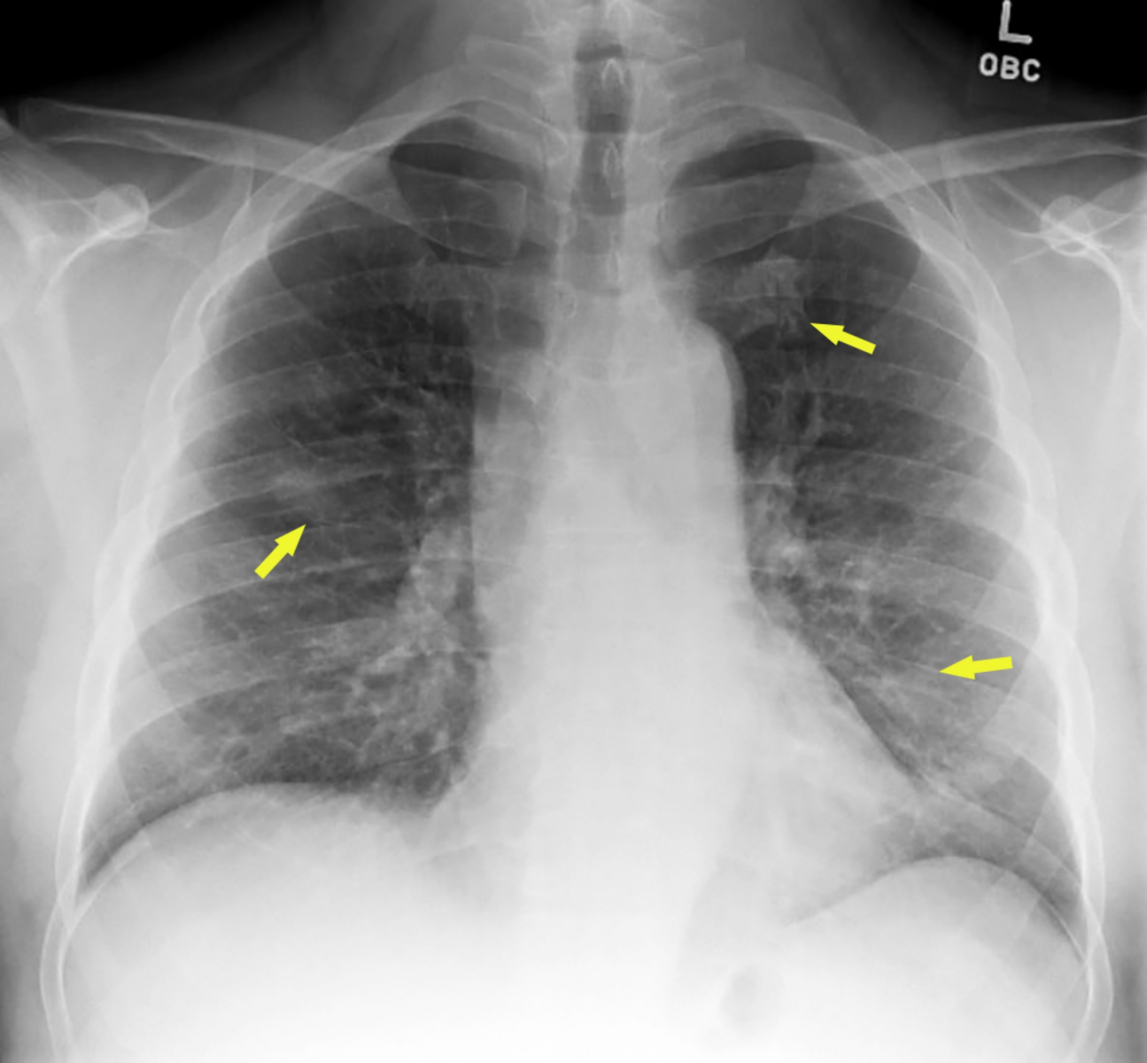
Chest X-ray showing hazy bilateral lung opacities (arrows)

Given his presentation and likely exposure, he was put on contact and droplet isolation in a negative pressure room. While more test results were awaited, he was started on empiric ceftriaxone and azithromycin for suspected community acquired pneumonia. Initial chest x-ray revealed hazy bilateral lung opacities (Figure 1).

**Figure 2:**
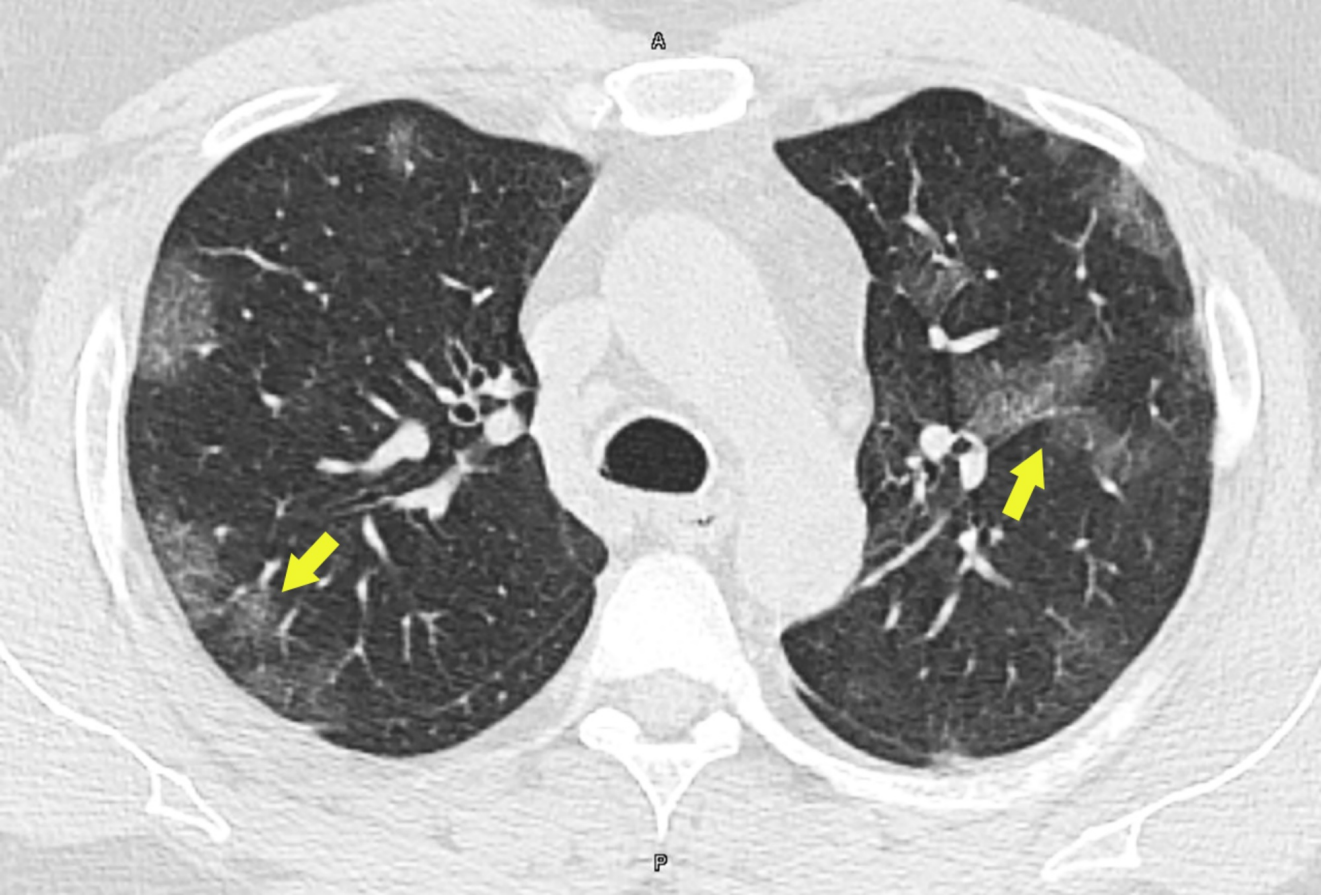
CT scan of thorax showing multifocal bilateral ground-glass lung opacities (arrows)

**Figure 3:**
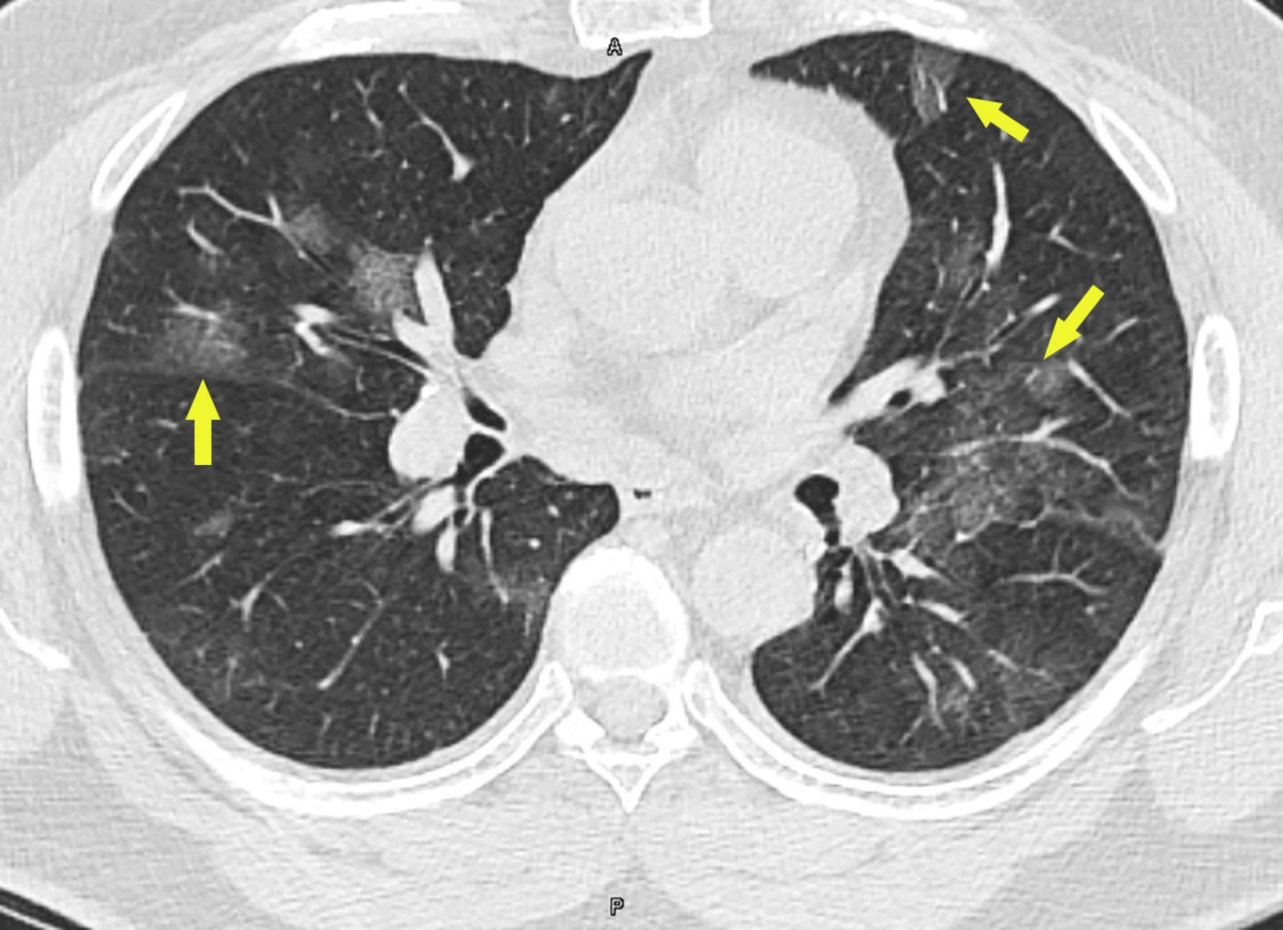
CT scan of the thorax showing multifocal bilateral ground-glass lung opacities with areas of subpleural sparing (arrows)

A CT scan of the thorax revealed multifocal bilateral ground-glass lung opacities with areas of subpleural sparing (Figures 2, 3).

Per institutional protocol, rapid influenza screen and respiratory viral panel were ordered, and both were negative. Thereafter, 2019-nCov testing (SARS-CoV-2 nucleic acid amplification [NAA]; LabCorp, Burlington, NC) was ordered. While results were awaited, the patient was shifted to the intensive care unit (ICU) due to worsening shortness of breath and oxygen requirement. High-flow nasal cannula (HFNC) support was provided, initially starting at 40 L/min flow and 100% supplemental oxygen. Over the next few days, HFNC was titrated down to 35 L/min flow and 40% supplemental oxygen. After the SARS-CoV-2 test came back positive, on the recommendations of pulmonary medicine and infectious disease teams we started oral hydroxychloroquine (Plaquenil) 200 mg twice daily for five days and discontinued azithromycin and ceftriaxone. After the third day on hydroxychloroquine, his fever has subsided and the patient started feeling better. As the patient continues to improve, HFNC continues to be downtitrated and further disposition is being planned.

## Discussion

SARS-CoV-2 is a beta coronavirus, with enveloped positive sense ribonucleic acid, in family Coronaviridae, subgenus Sarbecovirus, and Orthocoronavirinae subfamily [2,6]. It is the seventh coronavirus with human susceptibility [2,3]. Coronaviruses generally attack the respiratory tract. Though most infections are mild, some like SARS-CoV and MERS-CoV can lead to severe and potentially fatal infections.

COVID-19 infections have been noted to be more common in men; these can range from mild to severe and are often related to underlying medical conditions of the host including hypertension, diabetes mellitus, chronic obstructive pulmonary disease, cardiovascular and cerebrovascular diseases [1,2,7]. Though SARS-CoV-2 was first traced to a seafood market in Wuhan, China, the source is not yet clear. Bats are considered natural reservoirs, and pangolins may also be involved [2,6]. In retrospect, SARS-CoV had spread from palm civets and MERS-CoV from dromedary camels [2,6].

It is now clear that SARS-CoV-2 attaches to the angiotensin-converting enzyme 2 receptor in the respiratory epithelium to attach to and infect cells [1,2]. It is very infectious with a low infective dose and can be transmitted by droplets, direct contact and via respiratory secretions and has also been isolated in fecal swabs. The incubation period varies from one to fourteen days with a median of three to seven days [2]. COVID-19 presentation may vary from a mild illness to pneumonia and even septic shock with acute respiratory distress syndrome [1,2,5,7]. Cytokine storm due to excessive elaboration of tumor necrosis factor-alpha (TNF-α) and interleukins (IL)-2, IL-6, IL-7, and IL-10 has been seen [1,2]. It commonly presents with fever (88%), cough (67.8%), fatigue (38.1%), sputum production (33.4%), dyspnea (18.6%), sore throat (13.9%), headache (13.6%) and sometimes even with vomiting (5%) and diarrhea (3.8%) [2]. Lymphopenia is common [1,2]. CT of the thorax commonly reveals bilateral multilobar ground-glass opacities with peripheral or posterior distribution, with consolidation seen more in the elderly [5,8]. The radiological features peak around the 10th to 11th day of illness. Septal thickening, bronchiectasis, pleural thickening, subpleural involvement and cavitation are less common. Pleural and pericardial effusions are rare [8].

Diagnosis is generally performed from respiratory tract secretions via NAA testing as set forth by WHO, Centers for Disease Control and Prevention (CDC) and Infectious Disease Society of America guidelines [3,7,9]. In the absence of proven antivirals against SARS-CoV-2, treatment is generally symptomatic along with respiratory support. In patients with refractory hypoxemia, WHO has recommended the use of extra corporeal membrane oxygenation. Many drug trials are underway and a few have been promising [2,3,7,9]. The first US patient was successfully treated with remdesivir [4]. Hydroxychloroquine has been shown to alter the pH of the cell membrane, thus interfering with viral fusion [2,10]. It also inhibits viral nucleic acid replication, glycosylation of viral proteins, viral assembly and release [10]. Furthermore, it has immunomodulatory effects, suppressing TNF-α and IL-6 [2,10].

The CDC has been guiding US healthcare providers on the care of COVID-19 patients. However, in the setting of a pandemic, it is of utmost importance to be able to sever the chain of transmission so that new cases can be reduced and allow the healthcare delivery system to catch up.

## Conclusions

COVID-19 has rapidly become a pandemic in only months due to the easy transmissibility of the SARS-CoV-2 virus aided by a huge number of mildly symptomatic patients. However, it is severe and life-threatening in older individuals and those with underlying conditions. With no proven treatment at present, the outcome is generally poor for such patients. Early diagnosis and self-isolation or quarantine remain key to stemming the tide of the contagion as there is a real risk of the healthcare system being overwhelmed by the sick population.
